# Ibrutinib in B-cell lymphoma: single fighter might be enough?

**DOI:** 10.1186/s12935-020-01518-y

**Published:** 2020-09-29

**Authors:** Chao Xue, Xin Wang, Lingyan Zhang, Qingyuan Qu, Qian Zhang, Yujie Jiang

**Affiliations:** 1 Department of Hematology, Shandong Provincial Hospital, Cheeloo College of Medicine, Shandong University, Jinan, 250021 China; 2 School of Medicine, Shandong University, Jinan, 250012 Shandong China; 3 Department of Hematology, Shandong Provincial Hospital Affiliated to Shandong First Medical University, No.324, Jingwu Road, 250021 Jinan, Shandong China

**Keywords:** B-cell lymphoma, B cell receptor (BCR) signaling pathway, Bruton’s tyrosine kinase (BTK), Ibrutinib, Monotherapy

## Abstract

**Background:**

In recent years, the B cell receptor (BCR) signaling pathway has become a “hot point” because it plays a critical role in B-cell proliferation and function. Bruton’s tyrosine kinase (BTK) is overexpressed in many subtypes of B-cell lymphoma as a downstream kinase in the BCR signaling pathway. Ibrutinib, the first generation of BTK inhibitor, has shown excellent antitumor activity in both indolent and aggressive B-cell lymphoma.

**Main body:**

Ibrutinib monotherapy has been confirmed to be effective with a high response rate (RR) and well-tolerated in many B-cell lymphoma subgroups. To achieve much deeper and faster remission, combination strategies contained ibrutinib were conducted to evaluate their synergistic anti-tumor effect.

**Conclusions:**

For patients with indolent B-cell lymphoma, most of them respond well with ibrutinib monotherapy. Combination strategies contained ibrutinib might be a better choice to achieve deeper and faster remission in the treatment of aggressive subtypes of B-cell lymphoma. Further investigations on the long-term efficacy and safety of the ibrutinib will provide novel strategies for individualized treatment of B-cell lymphoma.

## Background

Among lymphatic malignancies, B-cell lymphoma is the most common type, accounting for 85% of non-Hodgkin lymphoma (NHL). It has been confirmed that the B cell receptor (BCR) signaling pathway, once revealed in 1993, plays an important role in the occurrence and development of B-cell lymphomas [1]. Bruton’s tyrosine kinase (BTK) is a downstream kinase that plays a central regulatory role in the BCR pathway [2]. Ibrutinib, the first-generation BTK inhibitor, has shown excellent antitumor activity in both indolent and aggressive B-cell lymphoma.

In recent decades, the efficacy and safety of ibrutinib monotherapy or combined with other agents have been explored in different subtypes of B-cell lymphomas [3, 4]. Even ibrutinib monotherapy has been the first-line treatment for some patients suffering symptomatic chronic lymphocytic lymphoma/small lymphocytic lymphoma (CLL/SLL) or elder/frail patients with primary central nervous system lymphoma (PCNSL) who can not endure high-dose methotrexate [5, 6]. Ibrutinib monotherapy might be enough for them to have a better life quality with well disease control. Furthermore, many researchers tried to combine BTK inhibitors with other agents to achieve deeper and faster remission. In this review, we will focus on the clinical progression and compare the efficacy of ibrutinib monotherapy or combination strategies for the treatment of B-cell lymphoma based on some ongoing or just-completed clinical trials.

## Bruton’s Tyrosine Kinase (BTK) and associated cross-linking signaling pathways

**Fig. 1:**
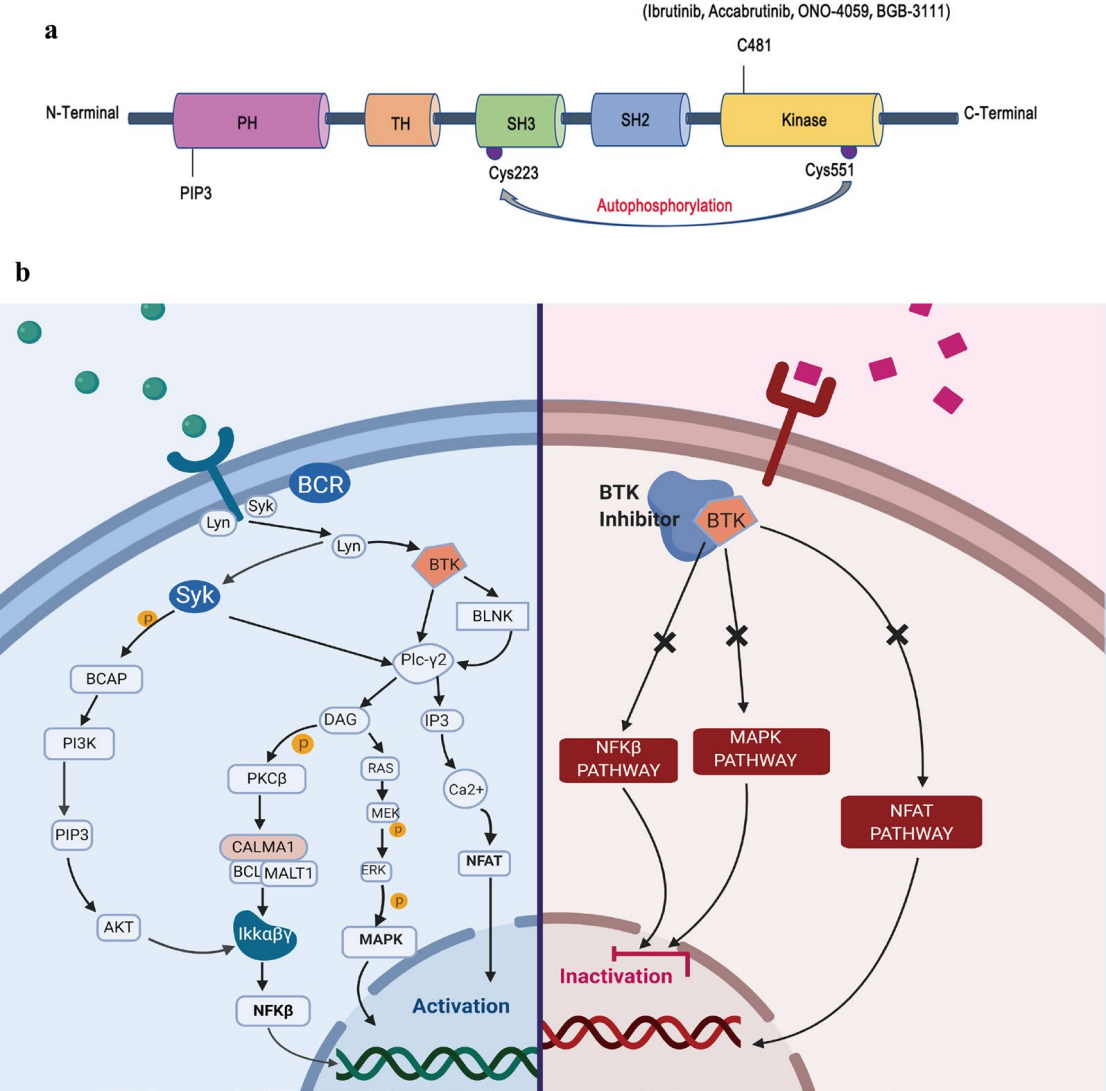
Structure of Bruton’s tyrosine kinase (BTK) and associated cross-linking signaling pathways. **a** BTK comprises five structural domains. BTK activation occurs twice during phosphorylation upon the plasma membrane. The first phosphorylation occurs on the Tyr551 site within the kinase domain by the Syk or Src family kinase, which subsequently leads to autophosphorylation of Tyr223 in the SH3 domain, achieving full activation of BTK kinase activity. **b** BTK activation process and inhibited result of cross-linking pathways by BTK inhibitors. The left figure shows when extracellular antigen bond BCR, BTK can regulate adverse cellular biological processes by activating multiple important pathways, such as NF-kB, MAPK, NFAT, and mTOR pathway. The right figure shows when the BCR pathway is irreversibly inhibited by small-molecule BTK inhibitors, its downstream pathway such as NF-kB, MAPK, and NFAT will also be inhibited, resulting in anti-tumor activity in B cell lymphoma

BTK is a nonreceptor tyrosine kinase of the TEC family that comprises five structural domains. It is continuously activated in B-cell lymphoma as a key mediator in tumor cell survival [7]. Following antigen binding to the extracellular part of BCR, activated BTK plays an essential signaling role in the BCR downstream pathway, which can regulate multiple cellular proliferation, differentiation, and apoptosis functions by cross-linking, activating many crucial proteins and pathways. These small-molecule BTK inhibitors irreversibly block its enzymatic activity by covalently bonding to the particular Cys-481 within the ATP binding pocket of BTK (Fig. 1a). BTK inhibitors not only inhibit the BCR signaling pathway, but also inhibit other downstream pathways including NF-kB, MAPK, NFAT, and mTOR pathways. The inhibition of those cross-linking pathways results in the activation of antitumor T cells and eventually the tumor eradication (Fig. 1b). Overall, BTK inhibitors involved not only in the BCR pathway but also in multiple important signaling pathways that closely related to B cell proliferation. This might interpret its powerful and high-effective inhibition effect in the treatment of B-cell lymphoma.

## Ibrutinib monotherapy in B-cell lymphoma

As a first-generation BTK inhibitor, ibrutinib was rapidly approved by the US Food and Drug Administration (FDA) for the treatment of CLL/SLL and MCL (mantle cell lymphoma) in 2014 and 2013, respectively. It was continuously approved for use as a single agent in patients with lymphoplasmacytic lymphoma (LPL)/WM (Waldenstrom’s macroglobulinemia) and marginal zone lymphoma (MZL) in 2015 and 2017, respectively [8]. Ibrutinib monotherapy exhibits a powerful anti-tumor effect and this magical small-molecular drug was honored with the “Prix Gallen Award” in 2015. In some subtypes of B-cell lymphoma, ibrutinib monotherapy has been confirmed to be enough to induce a satisfying response rate (RR). Here, we summarized the results of the available completed or ongoing clinical/preclinical trials of ibrutinib monotherapy (Table 1).

**Table 1:** Clinical trials of ibrutinib monotherapy in B-cell lymphoma

Disease	Study (year)	Phase	Study details	Efficacy	Refs.	
TN CLL	Byrd et al. (2018)	Ib/II	7-year follow up, 31 pts	ORR 87%, PFS 80%, OS 75%	[13]	
	O’Brien et al. (2014)	Ib/II	31 elderly pts	Objective response 71%, CR 13%, PR 55%	[15]	
	Burger et al. (2015)	III	269 elderly pts to compare ibrutinib with chloramnucil	ORR (86% vs. 35%), 18.4-month PFS (not reached vs. 18.9 months); 24-month OS (98% vs. 85%)	[39]	
R/R CLL	Byrd et al. (2018)	Ib/II	7-year follow up, 101 pts	ORR 89%, PFS 32%, OS 52%	[13]	
	Jones et al. (2018)	III	230 pts with del17p	30-month PFS 57%, OS 69%	[1]	
	Byrd et al. (2013)	Ib/II	85 pts, the majority of whom were considered to have high-risk disease, 51 received 420 mg, 34 received 840 mg	ORR (71% vs. 71%), PR with lymphocytosis (20% vs. 15%), 24-month OS and PFS were 83% and 75%	[11]	
	O’Brien et al. (2016)	II	145 pts with del17p	ORR 83%, 26-month PFS 63%, 26-month OS 75%,	[12]	
	Byrd et al. (2014)	III	391 pts at risk for a poor outcome, to compare ibrutinib with ofatumumab	ORR (42.6% vs. 4.1%), PFS 88% and PR with lymphocytosis of ibrutinib group was 20%, 12-month OS (90% vs. 81%)	[14]	
R/R MCL	Jeon et al. (2019)	NA	33 pts	ORR 33%, OS 35.1 months, PFS 27.4 months	[21]	
	Wang et al. (2015)	II	Long-term follow up of 111 pts, at a dose of 560 mg once daily	ORR 67%, CR 23%, 24-month OS 47%, 24-month PFS 31%	[17]	
	Dreyling et al. (2016)	III	280 pts to compare ibrutinib (n = 139) with temsirolimus (n = 141)	ORR (72% vs. 40%), CR with ibrutinib was 19%, median PFS (14.6 vs. 6.2 months), OS (not reached vs. 21.3 months)	[19]	
R/R FL	Advani et al. (2013)	I	56 pts	ORR 38%	[29]	
	Bartlett et al. (2017)	II	40 pts with ibrutinib 560 mg once daily	ORR 37.5%, CR 12.5%, 2-year PFS 20.4%, median PFS 14 months	[30]	
R/R ABC-DLBCL	Wilson et al. (2017)	I/II	38 pts	CR or PR 37%	[32]	
PCNSL	Grommes et al. (2017)	I	13 pts	ORR 77%	[34]	
	Lionakis et al. (2017)	Ib	18 pts	PR 83%	[35]	
R/R WM	Dimopoulos et al. (2016)	III	31 rituximab- refractory pts with a dose of 420 mg	ORR 90%, MRR 71%, OS 97%, PFS 86%	[44]	
	Treon et al. (2015)	II	63 pts with ibrutinib 420 mg once daily	ORR 90.5%, MRR 73%, PFS 69.1%, OS 95.2%	[25]	
R/R MZL	Noy et al. (2016)	II	63 pts with ibrutinib 560 mg once daily	ORR 48% (6 CRs, 26 PRs)	[28]	

Pts, Patients; DLBCL, Diffuse large B-cell lymphoma; ABC, Activated B-cell like; non-GCB, Germinal center B cell-like; CLL/SLL, Chronic lymphocytic leukemia/small lymphocytic lymphoma; WM, Waldenstroms macroglobulinemia; FL, Follicular lymphoma; MCL, Mantle cell lymphoma; MZL, Marginal zone lymphoma; PFS, Progression-free survival; PR, Partial response; ORR, Overall response rate; MRR, Major response rate; R/R, Relapsed/refractory; TN, Treatment-native; del17p, 17p deletion; At risk for a poor outcome, A short duration of response to therapy or adverse cytogenetic abnormalities; High-risk, Pts with del17p, del11q or with TP53 mutations.

### *Ibrutinib monotherapy in relapsed/refractory (R/R)* *and* *treatment-native (TN) CLL/ SLL*

Before the ibrutinib era, monotherapy strategies for the treatment of CLL/SLL include chlorambucil, rituximab, or bendamustine. Rituximab combined with the nucleoside analog (fludarabine) and cyclophosphamide (R-FC) regimen has been the standard treatment for the patient with CLL/SLL who meet the criteria to start treatment [9]. Although long-term disease-free survival (DFS) and durable remission can be achieved in many patients, the prognosis of some elderly patients or patients with deletion 17p (17p−) is still unsatisfactory due to chemotherapy-related toxicity and poor response to the above agents. The accurate mechanism for the development of R/R CLL/SLL is still unknown and 17p− has been recognized as the most important adverse prognostic factor [10, 11]. In the pre-ibrutinib era, the overall survival (OS) of patients with 17p− is only approximately 38%. Allogeneic stem cell transplantation (allo-SCT) was performed in an eligible patient. However, transplantation-related morbidity (TRM) remains an obstacle to achieving long-term survival. Some elderly or frail patients have no chance of receiving allo-SCT.

The occurrence of ibrutinib significantly changed this situation and the data came from real-world settings indicated that ibrutinib can overcome the adverse effect of the p53 mutation to some extent [12]. The possible reason to interpret the mechanism of ibrutinib on the p53 mutation might be that ibrutinib does not require a fully functional p53 pathway. In other words, ibrutinib plays its role regardless of p53 mutation status [4]. Until 2019, the largest scale study about ibrutinib in patients with 17p− was reported by Jones and his colleagues. They evaluated the outcomes in 230 patients with R/R 17p- CLL/SLL from three ibrutinib studies. With a median follow-up of 28 months, OS was 57%, the estimated 30-month progression-free survival (PFS) and OS were 57% and 69%, respectively [1]. Another study reported by Byrd et al. described the efficacy of ibrutinib monotherapy of the 7-year follow-up in R/R CLL/SLL patients with other unfavorable cytogenetic mutations [13]. Among 132 patients, 101 were R/R disease with del 17p (34%), del 11q (35%), del 13q (47%), and unmutated IGVH (78%), respectively. A durable response with an overall response rate (ORR) of 89% was maintained in all the patients and the median duration of response was 57 months in R/R patients, providing the evidence overcoming the influence of adverse cytogenetic abnormalities with ibrutinib. Furthermore, Byrd et al. conducted a controlled phase 3 study to evaluate the efficacy of ibrutinib in CLL/SLL patients at risk for a poor outcome (a short duration of response to efficacy or adverse cytogenetic abnormalities) by comparing ibrutinib with ofatumumab (a humanized anti-CD20 monoclonal antibody). The data indicated that ibrutinib has significantly higher single-agent efficacy in contrast to ofatumumab (ORR 42.6% vs. 4.1%, OS 90% vs. 80%) [14].

Based on the previous clinical data in R/R CLL/SLL, ibrutinib monotherapy is more effective and tolerant compared with other agents. Therefore, many researchers tried to use ibrutinib in patients with TN CLL/SLL. In a phase 1b/2 study, ibrutinib monotherapy was given to patients with TN CLL/SLL aged at least 65 years old [15]. The patients received 28-day cycles of once-daily ibrutinib at a dose of 420 mg or 840 mg. Seventy percent (22/31) patients achieved an objective response, including 4 complete response (CR), 1 nodular partial response, and 17 partial response (PR). Another phase 3 trial was conducted by Beuger and his colleagues comparing ibrutinib with chlorambucil in patients with TN CLL/SLL [3]. Their results indicated that compared with chlorambucil, ibrutinib showed a significantly superior in terms of PFS, OS, and RR (not reached vs. 18.9 months, 98% vs. 85%, 86% vs. 35%), respectively. Therefore, ibrutinib monotherapy in TN CLL/SLL also exhibited an effective and tolerable response as anticipated, especially in elderly patients.

Taken together, the numerous results from the existing clinical trials indicated that single-agent therapy with ibrutinib could be chosen as the first-line recommendation for both TN and R/R CLL/SLL patients with a durable response and well tolerance.

## Ibrutinib monotherapy in MCL

MCL is a heterogeneous subtype of NHL and some patients will relapse after short-term remission. With the improved understanding of molecular pathophysiology in MCL, some important signaling pathways, including BTK, NF-κB, and PI3K, have been extensively investigated in both MCL cell lines and biopsy samples of patients with MCL [16]. Until now, most of the clinical trials of ibrutinib in MCL were conducted in R/R disease. Wang et al. conducted a phase 2 study that enrolled 111 (median age of 68 years) patients with R/R MCL [17]. In this study, patients were enrolled into two sub-groups: those who had previously received ≥ 2 cycles and those received 0 ~ 1 cycle of bortezomib therapy. Single-agent ibrutinib was administered at a daily dose of 560 mg. The RR, CR, and PR was 68%, 21%, and 47%, respectively. They also observed that prior treatment with bortezomib had no effect on the RR and the estimated OS was 58% at 18 months [18].

Temsirolimus, an mTOR inhibitor, has been approved for the treatment of patients with R/R MCL in ESMO (European Society of Medical Oncology) guidelines. However, it is usually accompanied by adverse events (AEs) such as thrombocytopenia, anemia, fatigue, and diarrhea. Drying et al. conducted a phase 3 clinical trial to assess the efficacy and safety of ibrutinib *versus *temsirolimus in patients with R/R MCL [19]. A significantly longer PFS, ORR, and CR in ibrutinib than that in the temsirolimus group (14.6 months vs. 6.2 months, 72% vs. 40%, 19% vs. 1%) have been observed in the primary efficacy analysis. Also, ibrutinib was better tolerated than temsirolimus with fewer discontinuations owing to AEs (6% vs. 26%). In nowadays, more ongoing clinical trials are accessing ibrutinib’s efficacy as the first-line treatment in newly diagnosed MCL, especially for those ineligible for autologous stem cell transplantation (ASCT) at the first remission stage [20].

Unlike ibrutinib monotherapy in CLL/SLL, it might not be enough for patients with MCL. A real-world study indicated a median OS and PFS after ibrutinib monotherapy in R/R MCL patients were 35.1 months and 27.4 months, respectively [21]. This might be interpreted by the biological differences and more complex cross-action signaling pathways in MCL patients. Therefore, ibrutinib monotherapy might not be the best choice for this population and other choices such as combination strategies followed by stem cell transplantation might be favorable for them [21].

## Ibrutinib monotherapy in WM/LPL

Quite an amount of patients with WM/LPL can be employed “watchful waiting” until they meet the criteria to begin treatment, while some high-risk patients will refractory to rituximab or bortezomib contained chemotherapy regimen [22]. The myeloid differentiation primary response 88 (MYD88) mutation is highly prevalent in approximately 90% of patients with WM, which triggers the growth of tumor cells through BTK involved in the NF-κB pathway [23]. Therefore, ibrutinib will also theoretically be effective for patients with WM/LPL. A phase 3 clinical trial was conducted to assess the activity of ibrutinib in patients with rituximab-refractory WM [24]. As for 31 patients, most of them achieved a high ORR (90%), sustainable estimated median PFS (86%), and OS (97%). Many researchers predict that WM/LPL patients with the MYD88 mutation will benefit from ibrutinib compared with wild type MYD88. A report confirmed that patients with MYD88^wt^ and CXCR4^mut^ have a poor response to ibrutinib, however, the existing follow-up data show that some patients will still benefit from ibrutinib in the long-term, the accurate mechanism needed to be discussed in more studies [25–27]. Overall, preliminary data indicate that ibrutinib monotherapy is a potential new treatment choice for patients with newly diagnosed or R/R WM/LPL.

## Ibrutinib monotherapy in MZL and follicular lymphoma (FL)

MZL is a group of indolent B-cell lymphomas that originated from marginal zone B cells present in lymph nodes and extranodal tissues. MZL is associated with a variety of chronic infections, such as *Helicobacter pylori*, *hepatitis virus C, and parrot chlamydia*. Continuous antigen stimulation can activate the BCR signaling pathway, resulting in aberrant B cell abnormal hyperplasia and implicating BTK as a potential target in this malignancy. Ibrutinib may be an ideal candidate for MZL through blocking BTK, yielding a high clinical benefit rate, and clinically meaningful tumor shrinkage. The limited preliminary clinical results indicate a satisfactory profile of high ORR and durable responses. A multicenter, open-label, phase 2 study evaluated the efficacy and safety of ibrutinib in R/R MZL [28]. Among the 63 enrolled patients, PFS was 18 months after a median follow-up of 19.4 months, and the ORR was 51%. Subsequently, the outcomes were also analyzed by the MZL subtype. The median PFS was 13.8, 19.4, and 8.3 months for EMZL (extranodal MZL), SMZL (splenic MZL), and NMZL (nodal MZL), respectively. Therefore, ibrutinib was accelerated by the FDA for the treatment of R/R MZL in 2017, and it might be an effective, chemotherapy-free, targeted strategy option for this population.

Although typically indolent, FL remains an incurable disease. Some patients with FL will develop R/R disease and even transform into aggressive subtype NHL, such as diffuse large B-cell lymphoma (DLBCL) [29]. Based on the encouraging experience with other B-cell lymphomas, researchers applied ibrutinib to the treatment of FL. However, single-agent ibrutinib is not promising in both untreated or R/R FL patients. In a phase 2 study in R/R FL, ibrutinib produced an ORR 37.5%, CRR 12.5%, and a 2-year PFS 20.4%, these data showed no significant benefit compared with R-CHOP regimen [30]. The reason for the inefficiency of ibrutinib monotherapy in patients with FL remains unclear. The malignant cell origin, BCR signaling inhibition site, and other signaling pathways involved in the pathogenesis and development of FL might explain this result. Until now, rituximab contained regimen or rituximab maintenance is still recommended as the first-line treatment choice for FL patients. So more randomized pilots are needed to evaluate the potency of BTK inhibitors for patients with FL. Several explorations of BTK inhibitors for the treatment of FL are underway and a definitive conclusion has not been drawn yet.

## Ibrutinib monotherapy in DLBCL

DLBCL is the most common subtype of B-cell malignancy, accounting for 30 to 40% of all cases. Activated B cell-like (ABC) and germinal center B cell-like (GCB) are two major subtypes of DLBCL that are induced by distinct mechanisms. The prognosis of the ABC subtype is usually worse than that of GCB. However, patients in the ABC type can benefit from ibrutinib because malignant B cells in the ABC subtype selectively acquire mutations by targeting the BCR to foster chronic active BCR signaling [31]. In a phase 1/2 clinical study in which 20 patients were enrolled, ibrutinib monotherapy produced RR in 37% of ABC cases but only 5% in patients with GCB-DLBCL. Furthermore, the most noticeable RR (80%) was observed in tumors with concomitant MYD88 and BCR-associated protein CD79B mutations. This is consistent with the response of ibrutinib in LPL/WM. However, this does not explain the whole situation. A higher response also occurred within ABC tumors that lacked BCR mutations, suggesting that oncogenic BCR signaling in ABC might not require BCR mutations and may be initiated by nongenetic mechanisms [32]. Nevertheless, the accurate mechanism of BTK inhibitor resistance and less response in other subtypes of DLBCL need further investigation.

Primary or secondary central nervous system lymphoma (PCNSL/SCNSL) is a rare subtype of extranodal lymphoma, with a very poor prognosis and a median survival of only 2 months without additional treatment. Most of the histological types of PNCSLs are DLBCL and high-dose methotrexate (MTX)-based regimens are recognized as the first-line treatment choice [33]. However, the cure rate remains below 40%, and the tumor is prone to late recurrences. When recurrences develop, the patients often fail to respond to the former therapy and progress quickly. Novel insights into the pathogenesis of PCNSL indicate that PCNSL harbors mutations of hyperactive BCR signaling. Grommes et al. performed a phase 1 clinical study to evaluate the tolerability of ibrutinib monotherapy in 20 patients with R/R CNSL [34]. A total of 77% (10/13, 5 CR, 5 PR) of patients with PSCNL and 71% (5/7, 4 CR) of patients with SCNSL represented a clinical response. The median PFS was 4.6 and 7.43 months in PCNSL and SCNCL, respectively. Their clinical data and genomic analysis indicated that the differences between the two types might be due to the distinguishing features of BTK dependence and BCR pathway mutations. Interestingly, this study also found that the anti-tumor activity of ibrutinib in R/R PCNSL is much higher than that in patients with R/R DLBCL outside the CNS (ORR 77% vs. 25%, OS 15 months vs. 6.4 months). This may be because MYD88 and CD79B mutations are more common in PCNSL than in DLBCL outside the CNS, and the brain microenvironment might promote BTK dependence through chronic antigen expression and BTK activation. Meanwhile, these data suggest that further studies are needed to determine how genetic and tumor microenvironment factors, alone or in combination, create intrinsic BTK dependence in different B-cell lymphoma. Subsequently, a proof-of-concept phase Ib study was established to evaluate the efficacy and safety of ibrutinib monotherapy followed by ibrutinib plus chemotherapy (DA-TEDDi-R) in this population [35]. Their results indicated that ibrutinib appeared to enhance the efficacy of chemotherapy and significantly improve the outcomes of PCNSL patients.

## Combination therapy studies contained ibrutinib for B-cell lymphoma

Although ibrutinib monotherapy has potent efficacy and durable response in many subtypes of B-cell lymphoma, patients who can achieve a faster, deeper, and durable CR remain a minority, especially in some aggressive cases. Ibrutinib combination regimens are undergoing exploration, however, the optional combination strategies remain controversial [36–38]. Table 2 lists the current results of various combination strategies contained ibrutinib in B-cell lymphoma.

**Table 2:** Clinical trials of ibrutinib combination strategies in B-cell lymphoma

	Therapeutic regimens	Study (year)	Disease	Phase		Study details	Efficacy	Refs.	
*Ibrutinib-Anti-CD20 Monoclonal Antibody*	Ibrutinib-Rituximab (IR)	Burger et al. (2019)	R/R CLL	II		40 pts with high-risk CLL	36-month PFS 86.9%	[39]	
		Burger et al. (2019)	CLL	NA		208 pts, 181 pts with R/R CLL, 27 TN pts with high-risk disease	36-month ORR 92%, PFS 86.9%	[34]	
		Jain et al. (2017)	R/R MCL	II		50 pts	ORR 88%, CR 58%, PR 30%	[42]	
		Dimopoulos et al. (2018)	WM	III		150 pts	MRR 72%, 30-month PFS 82%	[44]	
	Ibrutinib-Obinutuzumab	Moreno et al. (2019)	TN CLL	III		116 pts	ORR 88%, CR 19%, 30-month PFS 79%	[36]	
	Ibrutinib-Ofatumumab	Jaglowski et al. (2015)	CLL	Ib/II		66 pts, ibrutinib lead-in (group 1), concurrent start (group 2), or ofatumumab lead-in (group 3)	ORR were 100%, 79% and 71%; 12-month PFS 89%, 85%, 75% in group1 ~ 3, respectively	[37]	
	Ibrutinib-Ublituximab	Sharman et al. (2017)	R/R CLL	II		45 pts	6-month ORR 88%, pts with high-risk ORR 95%	[38]	
*Ibrutinib-Chemoimmunotherapy*	Ibrutinib-BR	Fraser et al. (2019)	R/R CLL	III		289 pts	36-month PFS 68%, OS 81.6%	[45]	
		Brown et al. (2015)	R/R CLL	Ib		30 pts	ORR 93.3%, CR 40%, OS 74%, PFS 86.3%	[46]	
		Chanan-Khan et al. (2016)	R/R CLL	III		289 pts	ORR 83%, CR10%, PFS 79%	[48]	
	Ibrutinib-FCR	Davids et al. (2019)	CLL	II		85 pts, 5% del17p, 4% TP53 mutations, 2 pts with both	16.5-month CR 33%	[42]	
		Brown et al. (2015)	R/R CLL	Ib		3 pts	ORR 100%, CR 67%, PFS 70.3%	[46]	
		Shanafelt et al. (2019)	CLL	III		354 pts to IR group, 175 pts to the FCR group	33.6-month PFS 89.4% vs. 72.9%; OS 98.8% vs. 91.5%	[48]	
	Ibrutinib-R-ICE	Sauter et al. (2018)	DLBCL	I		21 pts	ORR 90%, CR 55%, PR 35%	[49]	
	DA-TEDDI-R	Lionakis et al. (2017)	PCNSL	Ib		18 pts	ORR 86%	[35]	
	Ibrutinib-R-CHOP	Younes et al. (2014)	NHL	Ib		32pts	ORR 94%	[50]	
	Ibrutinib-R-HD-MTX	Grommes et al. (2019)	R/R CNSL	Ib		15 pts	19.7-month ORR 80%, CR 53.3%, PR 26.7%	[51]	
*Ibrutinib-Biotarget Agents*	Ibrutinib-Rituximab-Lenalidomide	Ujjani et al. (2018)	R/R CLL	I	12 pts		ORR 67%, 12-month PFS 83%	[62]	
		Jerkeman et al. (2018)	R/R MCL	II	50 pts		ORR 76%, CR 56%, PR 20%	[2]	
		Ujjani et al. (2016)	FL	I	22 pts		ORR 95%, 12-month PFS 80%	[63]	
	Ibrutinib-Venetoclax	Jain et al. (2019)	TN CLL	II	80 pts (untreated high-risk and the median age was 65 years)		CR or CR with incomplete count recovery 88%; 1-year PFS 98%, OS 99%	[60]	
		Tam et al. (2018)	R/R MCL	II	24 pts, 50% with TP53 mutation, 75% had high-risk prognostic score		CR 42%	[54]	
	Ibrutinib-Venetoclax-Obinutuzumab	Rogers et al. (2018)	R/R CLL	Ib	12 pts		ORR 92%, CR 42%	[55]	
	Ibrutinib-Palbociclib	Martain et al. (2019)	MCL	I	27 pts		25.6-month ORR 67%, CR 37%, PFS 59.4%	[54]	
	Ibrutinib-Umbralisib	Davids et al. (2019)	R/R CLL	Ib	21 elder pts with more than two previous therapies		ORR 90%, CR 29%, PR 62%	[57]	
		Davids et al. (2019)	R/R MCL	Ib	21 pts, median age of 68 years		ORR 67%, CR 19%, PR 48%	[57]	
	Ibrutinib-Umbralisib-Ublituximab	Nastoupil et al. 2019)	CLL	I	46 pts		ORR 84%	[58]	
	Ibrutinib-Nivolumab	Younes et al. (2019)	NHL	I/2a	141 pts		ORR, high-risk CLL/SLL 61%, FL 33%, DLBCL 36%	[59]	

Pts, Patients; CLL/SLL, Chronic lymphocytic leukemia/small lymphocytic lymphoma; WM, Waldenstroms macroglobulinemia; MCL, Mantle cell lymphoma; MZL, PFS, Progression-free survival; PR, partial response; CR, Complete response; ORR, Overall response rate; MRR, Major response rate; OS, Overall survival; R/R, Relapsed/refractory; TN, Treatment-native; FCR, Fludarabine combined with cyclophosphamide and rituximab; R-ICE, Rituximab, ifosfamide, carboplatin, etoposide; R-CHOP, Rituximab plus cyclophosphamide, doxorubicin, vincristine, prednisone; R-HD-MTX, High-dose methotrexate; High-risk, Pts with del17p, del11q or with TP53 mutations

## Ibrutinib with anti-CD20 monoclonal antibody and chemoimmunotherapy

In the beginning, many researchers combined rituximab (the first-generation anti-CD20 monoclonal antibody) with ibrutinib to investigate if the patients with CLL/SLL can benefit from this combination therapy. Burger et al. conducted a randomized single-center trial of ibrutinib single-agent *versus* ibrutinib plus rituximab (IR) in 208 (181 R/R and 27 NT diseases) patients with high-risk ( 17p- or TP53 mutation) CLL. After a median follow-up of 36 months, the PFS was 86% and 86.9% in the ibrutinib and IR group, respectively. The results from subsequent multicenter trials also proved that IR could not improve PFS although these patients exhibited faster RR and low residual disease levels [39–41].

There were not many randomized and controlled studies comparing ibrutinib monotherapy or IR in MCL patients. Jain et al. reported a single-arm, phase II clinical trial of IR in patients with R/R MCL [42]. Twenty-nine of 50 (58%) achieved CR and those with low ki 67 patients had more durable remissions. This result is relatively satisfied compared with that of 40% in our previous section for patients with R/R MCL who received ibrutinib monotherapy. However, a credible and comprehensive conclusion about the pros and cons of ibrutinib monotherapy or IR can not be drawn unless validated by larger samples, an accurate prognosis score combined with molecular biological analysis has been done.

Although quite an amount of studies have confirmed that IR is superior to rituximab monotherapy for patients with WM/LPL, there are not many controlled or head to head pilots to evaluate the difference between IR and single-agent ibrutinib [43, 44]. However, ibrutinib is at least an ideal choice, especially for elderly patients or those who are not eligible for intensive chemotherapy. Furthermore, patients with WM/LPL often suffer from amyloidosis, poor prognostic comorbidity with a high mortality rate, these patients often have a very poor response and tolerance to intensive chemotherapy.

There are also some studies to assess the RR of the combined chemoimmunotherapy strategy contained or not contained ibrutinib [45–52]. In 2016, Chanank-Khan et al. compared the RR of ibrutinib combined with bendamustine and rituximab (IBR) and placebo-BR in 578 patients with R/R CLL/SLL (HELIOS trial) [53]. At a median follow-up of 17 months, PFS in the IBR group was significantly high than that of the placebo-BR group (not reached vs. 13.3 months). The AEs were similar in both groups. It is notable that patients with 17p- were excluded in the HELIOS trial. Although it seemed that the result from this randomized and double-blind trial indicated a significantly improved outcome with the IBR regimen, the data did not mean that IBR is superior to ibrutinib monotherapy because the molecular biological factors have not been incorporated into concern in this study. Therefore, ibrutinib monotherapy is still recommended as the first-line choice in patients with R/R or NT CLL/SLL.

We should also realize the importance to make a balance between clinical effectiveness and cost in some developing countries, especially in those both agents can not be covered by medical insurance. Based on the existing studies, ibrutinib combining rituximab or chemoimmunotherapy might be unnecessary for some indolent cases.

## Ibrutinib with other biotarget agents

As shown in Fig. 1b, BTK can regulate multiple cellular proliferation by activating multiple significant pathways, such as NF-kB, MAPK, NFAT, and mTOR pathway. It also indicated a cross-link of BCR-associated kinases (SYK, BTK, PKC, and PI3K) inhibitors. Therefore, if we combined ibrutinib with the above-associated bio target agents, a synergistic effect might be achieved [47, 54–59]. Here we chose CLL/SLL and MCL as examples and the comparison between ibrutinib monotherapy and combination regimens was listed in Table 3. Jain et al. conducted a preclinical investigation and confirmed that ibrutinib plus venetoclax (a BCL-2 inhibitor) had potential synergistic interaction as the first-line treatment for older patients with R/R CLL/SLL [60]. After 12 cycles of combined treatment, 88% of the patients achieved CR with undetectable minimal residual disease (MRD) and no added AEs. In addition to CLL/SLL, there were also quite a few studies in MCL and WM/LPL population which reported high RR with ibrutinib combined venetoclax [54, 61].

**Table 3:** The comparison of clinical trials of ibrutinib regimens in TN CLL and R/R MCL population

	Ibrutinib	Ibrutinib-Rituximab	Ibrutinib-Chemoimmunotherapy	Ibrutinib-Venetoclax
*TN-* *CLL*	*(Burger et al. 2015)* [3] 269 pts with a median age of 73 years; *Regimen*, ibrutinib 560 mg per day *Outcome*, 18.4-month PFS not reached, 2-year OS 98%, ORR 86%.	*(Shanafelt et al. 2019)8* [3] 354 pts aged 70 years or younger; *Regimen*, ibrutinib per day, 6 cycles rituximab; *Outcome*, 33.6-month PFS 89.4%, OS 98.8%; 3-year PFS among pts with IGHV mutation 87.7%.	*(Shanafelt et al. 2019)* [48] 175 pts aged 70 years or younger; *Regimen*, ibrutinib per day, 6 cycles of fludarabine, cyclophosphamide and rituximab; *Outcome*, 33.6-month PFS 72.9%, OS 91.5%; 3-year PFS among pts with IGHV mutation 88%.	*(Jain et al. 2019)* [60] 80 high-risk and older pts with CLL; *Regimen*, ibrutinib 420 mg once daily for 3 cycles, venetoclax (weekly dose escalation to 400 mg once daily); *Outcome*, CR 96% after 18 cycles, and 69% had remission with undetectable MRD; 1-year PFS and OS were 98% and 99%.

*R/R MCL*	*(Wang et al. 2013)*^*1*^*7* 111 pts with a median age of 68 years and 86% had intermediate or high-risk MCL; *Regimen*, ibrutinib 560 mg per day *Outcome*, ORR 68% (CR 21%, PR 47%); 15.3-month PFS was 13.9 months, 18-month OS 58%.	*(Wang et al. 2016)*^*41*^ 50 pts with the median number of previous regimens were three; *Regimen*, ibrutinib 560 mg per day, rituximab 375 mg/m2 once per week during cycle1, on day 1 of cycles 3–8, and thereafter once daily another cycle up to 2 years; *Outcome*, 16.5-month ORR 88%, CR 44%, PR 44%.	*(Karmali et al. 2019)*^*52*^ 36 pts with CR or PR to frontline chemo-immunotherapy +/- auto-SCT ; *Regimen*, Received I-M 560 mg/day for up to 4 years; *Outcome*, I-M is feasible in these pts with manageable toxicities related to the known safety profile of Ibrutinib, but the PFS and OS data are needed larger clinical studies to access.	*(Tam et al. 2018)*^*54*^ 24 pts with previously untreated MCL and 75% had high-risk prognostic score; *Regimen*, Ibrutinib 560 mg/day, venetoclax was weekly increasing dose to 400 mg/day after 4 weeks; *Outcome*, 16-week CR 42%, 78% of the pts with responses were estimated to have ongoing responses at 15 months.


Pts, Patients; I-M, Ibrutinib maintance; PR, Partial response; CR, Complete response; ORR, Overall response rate; R/R, Relapsed/refractory; TN, Treatment-native; AutoSCT, Autologous stem cell transplantation; MRD, Measurable residual disease; OS, Overall survival;

Lenalidomide has been proved to be effective in many subtypes of B-cell lymphoma [62, 63]. Jerkeman et al. combined ibrutinib, rituximab, and lenalidomide in the treatment of R/R MCL (PHLEMON trial) [2]. Before this trial, patients with R/R MCL were usually treated with high-dose cytarabine contained regimen plus rituximab and followed by ASCT or allo-HSCT. All three drugs were given 12 cycles of 28 days and ibrutinib plus rituximab were used as maintenance therapy. At a median follow-up of 17.8 months, 38 patients had an overall response, including 28 (56%) patients reached CR, and 10 (20%) reached PR. This promising result proposed “chemo-free” feasibility for patients with R/R MCL. In the future, the treatment mode of MCL might be significantly altered due to the satisfied efficacy of multiple small molecular bio-target drugs combination.

Overall, different from ibrutinib with chemotherapy, ibrutinib combined with other bio target agents exhibited a powerful synergistic anti-tumor effect in B-cell lymphoma. In the future, more prospective, randomized, and controlled trials are needed to evaluate the feasibility of a “chemo-free” strategy in real-world investigations.

## Conclusions

BTK is a central regulator of the BCR signaling pathway and targeting BTK has shown impressive efficacy in the treatment of various subtypes of B-cell malignancies. The advent of ibrutinib produced an epoch-making landscape with tolerated toxicity. Based on the existing studies, ibrutinib monotherapy has exhibited a powerful anti-tumor effect in almost all of the subtypes of B-cell lymphoma except for FL. Combination treatment contained ibrutinib has not been reached an agreement due to the chemotherapy-associated toxicity and the economic cost. In most of the investigations, it seems unnecessary to combine ibrutinib with the anti-CD20 antibody or chemotherapy, especially in indolent B-cell lymphoma. However, ibrutinib combined with other small molecular bio target agents might be a promising choice. In the future, more head-to-head comparisons and clinical trials are needed to assess the long-term efficacy and safety of the ibrutinib monotherapy or combination strategies to achieve much deeper and faster remission in the treatment of B-cell lymphoma.

## Data Availability

Not applicable.

## Abbreviations

BCR: B cell receptor
BTK: Bruton’s tyrosine kinase
RR: response rate
NHL: non-Hodgkin lymphoma
CLL/SLL: chronic lymphocytic lymphoma/small lymphocytic lymphoma
PCNSL: primary central nervous system lymphoma
FDA: Food and Drug Administration
MCL: mantle cell lymphoma
LPL: lymphoplasmacytic lymphoma
WM: Waldenstrom’s macroglobulinemia
MZL: marginal zone lymphoma
RR: response rate
TN: treatment-native
DFS: disease-free survival
OS: overall survival
Allo-HSCT: allogeneic stem cell transplantation
TRM: transplantation-related morbidity
PFS: progression-free survival
ORR: overall response rate
CR: complete response
PR: partial response
ESMO: European Society of Medical Oncology
AEs: adverse events
ASCT: autologous stem cell transplantation
MYD88: myeloid differentiation primary response 88
FL: follicular lymphoma
EMZL: extranodal MZL
SMZL: splenic MZL
NMZL: nodal MZL
DLBCL: diffuse large B-cell lymphoma
ABC: activated B cell-like
GCB: germinal center B cell-like
PCNSL/SCNSL: primary or secondary central nervous system lymphoma
MTX: methotrexate
IR: rituximab
MRD: minimal residual disease
